# Do Neighborhood Characteristics in Amsterdam Influence Adiposity at Preschool Age?

**DOI:** 10.3390/ijerph120505561

**Published:** 2015-05-22

**Authors:** E. Jessica Hrudey, Anton E. Kunst, Karien Stronks, Tanja G.M. Vrijkotte

**Affiliations:** Department of Public Health, Academic Medical Center, University of Amsterdam, Postbox 22660, 1100 DD Amsterdam, The Netherlands; E-Mails: a.kunst@amc.uva.nl (A.E.K.); k.stronks@amc.uva.nl (K.S.); t.vrijkotte@amc.uva.nl (T.G.M.V.)

**Keywords:** neighborhood characteristics, preschool age, adiposity, mixed models

## Abstract

Background: Neighborhood characteristics may contribute to adiposity in young children, but results in the current literature are inconsistent. This study aimed to investigate whether objective (socioeconomic status (SES)) and subjective (perceived safety, satisfaction with green spaces and perceived physical disorder) neighborhood characteristics directly influence child adiposity (as measured by BMI, percent body fat (%BF) and waist-to-height ratio (WHtR)). Methods: Data on child BMI, %BF and WHtR were obtained from the Amsterdam Born Children and their Development cohort at 5–6 years of age. Three thousand four hundred and sixty nine (3469) children were included in the analyses. Mixed models, using random intercepts for postal code area to account for neighborhood clustering effects, were used to analyze the relationships of interest. Results: Associations were observed for both perceived safety and neighborhood SES with %BF after adjustment for maternal education and ethnicity. All relationships were eliminated with the inclusion of individual covariates and parental BMI into the models. Conclusions: In general, child adiposity at age 5–6 years was not independently associated with neighborhood characteristics, although a small relationship between child %BF and both neighborhood SES and perceived safety cannot be ruled out. At this young age, familial and individual factors probably play a more important role in influencing child adiposity than neighborhood characteristics.

## 1. Introduction

In Europe in 2010, 33% of 6–9 year olds were estimated to be overweight or obese [1]. In the Netherlands the prevalence of overweight in 5–6 year old children nearly tripled between 1980 and 2009 [2,3]. Excessive weight gain early in life is associated with increased risks for overweight and obesity later in life, which in turn, increases the risk for developing chronic diseases such as diabetes or cardiovascular disease [4,5,6]. Preventing and reducing overweight in young children is, therefore, an essential goal for public health.

Although the cause of overweight can be simplified to an imbalance in energy intake and output, its development in preschool children is also a product of both familial and environmental influences. Young children are highly influenced by their parents and they have been shown to model their parents’ eating and activity behaviors [7,8,9]. Neighborhood characteristics may also influence adiposity in young children given that the prevalence of child overweight has been observed to be unevenly distributed across neighborhoods [10] and because neighborhood characteristics have been related to adiposity in adults and adolescents [11,12]. 

Knowing whether neighborhood factors directly influence a child’s adiposity, as well as which neighborhood characteristics are particularly important, is necessary to inform community-level policies targeting child overweight. However, it is possible that the relationships between neighborhood factors and child adiposity only reflect an indirect effect: neighborhoods may contribute to overweight in adults, who then raise children who become overweight. Moreover, the uneven prevalence of elevated child adiposity across neighborhoods may simply be a result of neighborhood composition, such as differences in the number of low socioeconomic status (SES) residents and non-Western ethnic groups.

Results from the current literature are inconsistent regarding the associations between specific neighborhood factors and adiposity in young children. Neighborhood SES appears to be related to child overweight [13,14,15], although the relationship is less clear in young children [16]. Studies on relationships between adiposity in young children and other neighborhood characteristics, such as safety, green spaces, and physical disorder have produced mixed results [17,18,19,20,21,22,23,24]. 

This study aimed to investigate whether neighborhood factors directly influence a child’s adiposity by analyzing the relationships between several neighborhood characteristics and adiposity in 5–6 year old children, as indicated by BMI, percent body fat (%BF) and waist-to-height ratio (WHtR). If a direct influence of neighborhood characteristics on child adiposity is observed, this could inform policy makers on how to modify the neighborhood context to help minimize unhealthy child adiposity.

This study complements the current literature in three respects. Firstly, with a few exceptions [25,26,27,28], most of the research to date has focused on BMI or overweight status in children as an outcome. However, %BF and WHtR are better predictors of future chronic disease risk [29,30,31,32]. Secondly, much of the research on child adiposity and neighborhood factors has been conducted in North America [7]. Results may differ across countries, particularly due to differences in cultural values, public transport systems and land-use [33,34]. Therefore, research in other settings is warranted. Finally, the method of measuring neighborhood characteristics has been problematic in previous research. Objectively measuring safety, quality of green spaces and physical disorder does not adequately capture how residents perceive their neighborhood [17,35]. However, when these characteristics are measured subjectively they are often based on the opinions of individual study participants, which only recognizes individual perceptions rather than the perceptions of the entire community [18,19,20]. This study instead measured community perceptions to identify subjective characteristics that are problematic on a neighborhood-wide level. 

## 2. Methods

### 2.1. Data Sources

Data were obtained from the ABCD cohort, a prospective, longitudinal birth cohort that began in 2003 [36]. Women (N = 8266) participated by completing a questionnaire during pregnancy. Of these women, 7863 gave birth to live singleton infants and 6575 agreed to follow-up and collection of growth data on their children. Data on the birth outcomes of these pregnancies were collected from the Youth Health Care (YHC) registry and 5218 mothers also completed a questionnaire once infants reached 3 months of age. Between 2008 and 2010, data on height, weight, waist circumference and %BF were collected from 3321 children during the ABCD health check. For those children who were not measured in this health check, but whose mothers consented to the collection of growth data, height and weight measurements were obtained from the YHC registry. These data were measured and documented by a Youth Health Care professional. Additionally, 4488 mothers completed questionnaires about their 5–6 year olds at this time. Written, informed consent was obtained from the mothers on behalf of their children and the study was conducted in accordance with the Declaration of Helsinki. The ABCD study (Project Number MEC02/039#02.17.392) and its informed consent procedures were approved by the Medical Ethics Review Committees of all medical centers in Amsterdam, by the Registration Committee of the Municipality of Amsterdam, and by the Central Committee on Research Involving Human Subjects in the Netherlands.

Neighborhood SES was determined using 2006 status scores obtained from the Social and Cultural Plan Bureau (SCP), a division of the Netherlands Institute for Social Research [37,38]. The remaining neighborhood characteristics (perceived safety, satisfaction with green spaces and perceived physical disorder) were obtained from the Dutch Housing Questionnaire (the WoON survey) for 2009 [39,40,41], a nationwide questionnaire on various environmental and neighborhood characteristics carried out by Statistics Netherlands (CBS). The WoON survey was completed by a study sample that was separate from the ABCD cohort.

### 2.2. Dependent Variables

Height of the children at age 5–6 years was measured to the nearest millimeter with a Leicester portable height measure (Seca, Hamburg, Germany) and weight was measured to the nearest 100 g with a Marsden M-4102 scale (Oxforshire, UK) [42]. BMIs were calculated from these measurements and were then converted into standard deviation scores (SDS). These SDS were corrected for child age at measurement and were derived from gender-specific reference curves based on ABCD study data. These reference curves were generated using the Lambda Mu Sigma method and were fitted using a Box-Cox power formula [43,44]. Waist circumference was measured to the nearest millimeter using a Seca measuring tape halfway between the iliac crest and the costal margin and converted into WHtR [42]. After children had emptied their bladders, %BF was measured twice in a supine position using dual-frequency, tetrapolar, arm-to-leg bioelectrical impedance analysis (BIA) with the BodyStat 1500 MDD device (BodyStat Inc., Douglas, UK). Data on the procedure and validation of this measurement in 5–6 year old children has been previously described [42,45]. A recalibrated version of the Kushner equation was derived from the validation procedure and was utilized to measure %BF in the children [45].

### 2.3. Independent Variables: Individual and Family Level

Individual and family level covariates were chosen after a literature review for the most important determinants of overweight in young children [7,46]. Data on these covariates were obtained from the ABCD questionnaires and the YHC registry. Variables representing individual early life history of the children included maternal pre-pregnancy BMI, maternal smoking during pregnancy (yes, no), duration of exclusive breastfeeding (<3 months, 3–6 months, ≥6 months) and age at introduction of solid foods (<4 months, ≥4 months). Child age and gender were also included in the analyses of %BF and WHtR. Familial level variables included maternal education level, as an indicator of family SES (low (<5 years of education after primary school), mid (5–10 years of education after primary school), high (≥10 years of education after primary school)), ethnicity (Western, non-Western), maternal BMI and paternal BMI. Ethnicity was based on the birth country of the child’s mother or maternal grandmother. Western ethnicity included Dutch or other European backgrounds and non-Western ethnicity included Turkish (5.9%), Moroccan (9.9%), Antillean (1.1%), Ghanaian (1.9%) Surinamese-African (3.7%) and Surinamese-Indian (2.0%) and other Surinamese (1.6%) backgrounds.

### 2.4. Independent Variables: Neighborhood Level

The children’s neighborhoods were defined as the four digit postal code areas that they were living in at the time of measurement. These areas are on average 2.5 km^2^ and they had a mean population of approximately 9329 residents in 2009 (interquartile range (IQR): 704–17954) [47,48]. Status scores were previously calculated by the SCP for each postal code area, using data from registries on the educational level, household income and employment status of area residents. Increasing status scores represented increasing neighborhood SES. The WoON data required additional refinement. Participants of the WoON survey were asked several questions about their neighborhood and provided opinions using a 5 point Likert scale (e.g., “I am satisfied with the green spaces in this neighborhood”). A single question from the survey was used for the variables perceived safety and satisfaction with green spaces. Physical disorder was constructed from four questions: presence of dog waste on the street, presence of graffiti, presence of litter and presence of vandalism. These variables were combined into a 10 point scale for physical disorder [41]. For each of these variables, we created a score for each postal code area by averaging individual responses from all WoON respondents in that area. For each WoON variable, an increasing score indicated increasing perceived safety, increasing satisfaction with green spaces and increasing physical disorder. If there were less than 15 respondents in a given postal code area, the data were excluded from the analyses. Of the areas included in the final study sample, there was an average of 54 respondents per postal code area (IQR: 14–94). There were three pairs of postal code areas that had too few children and too few WoON responses. The postal code areas from each pair were geographically adjacent and the WoON and status score data within each of these pairs were similar. Therefore, each pair was combined into one area and the WoON and status score data were averaged across the newly created area. 

### 2.5. Study Sample

**Figure 1:**
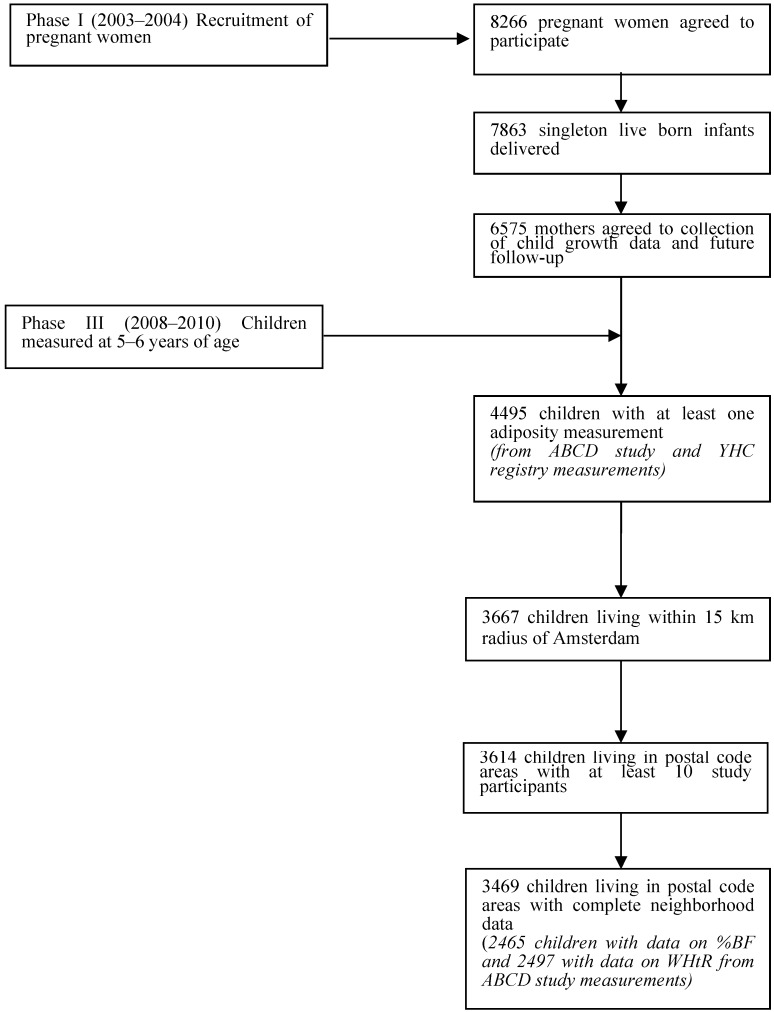
Study sample selection; ABCD=Amsterdam Born Children and Their Development Study, %BF = percent body fat, WHtR = waist-to-height-ratio, YHC = Youth Health Care.

From the 7863 singleton infants eligible for inclusion in this study, a total of 4495 children had data on height and weight measurements at 5–6 years of age, obtained via the ABCD health check or the YHC registry (a primary response of 57%). Children were excluded if there was no record of their postal code area at 5–6 years of age or if they did not live within a 15 km radius of Amsterdam. This reduced the sample to 3667 children. The number of children from the study sample living within each postal code area was then determined and any postal code areas with less than 10 children were excluded to ensure sufficient power for statistical analysis. This reduced the sample size to 3614 children. Finally, children were excluded if neighborhood data were missing on their postal code area, resulting in a final sample size of 3469 children (Figure 1). Of note, data on %BF and WHtR were only measured in children who attended the ABCD health check. There were 2465 children with data on %BF and 2497 children with data on WHtR in the final sample, but there remained a minimum of 10 children per postal code area with data for each of these outcomes.

### 2.6. Statistical Analysis

The distribution of the child outcomes and covariates of interest were analyzed across quartiles of each neighborhood characteristic. This was done to assess the distribution of child adiposity, as well as the other covariates of interest, across the characteristics of the neighborhoods. In order to test the influence of neighborhood characteristics on child adiposity, a mixed linear regression model was used for each environmental factor and each outcome (model 1), with the inclusion of random intercepts by postal code area to account for neighborhood clustering effects. For %BF and WHtR, age and gender were also included in this first model. Maternal education and ethnicity were added to model 1 to create model 2. These are familial factors that predate the birth of the child and thus are likely confounders. Therefore, model 2 would allow us to observe any associations after correction for confounding. In model 3, factors from the child’s early life history were included and finally in model 4, maternal and paternal BMI were included. The covariates in models 3 and 4 are possible mediators in the relationships, particularly if the family never moved between conception and the current measurement of the child. These covariates were included in separate models to avoid over correction of model 2 and to allow for investigation of possible mediating factors in the observed associations. 

We also extended the random structure of the models to investigate if potential explanatory variables act differently within clusters. These models were not found to fit significantly better than random-intercepts models and were, therefore, not used in the final analyses. Finally, intra-class correlation coefficients (ICC) were determined to investigate the extent to which child adiposity outcomes varied between neighborhoods. ICCs were calculated using only complete cases (*i.e.*, no missing data) to allow comparison between the ICC values as covariates were added to the models. Sensitivity analyses excluding the previously combined postal code areas and using the 2010 status scores rather than the 2006 status scores were completed to determine if the results would change substantively.

Outcomes were tested for normality and, if necessary, the best transformation was determined via the Box-Cox method. WHtR was found to be non-normal and an inverse transformation of this variable was applied. Both transformed and untransformed data were analyzed to ensure statistical robustness, but only the untransformed analyses were presented for clarity. This outcome was also multiplied by 100 to enhance readability because the differences for WHtR in young children are very small. A *p*-value < 0.05 was considered significant and all statistical analyses were completed using SPSS version 20 (SPSS Inc., Armonk, NY, USA) and R version 3.1.1 (R Foundation for Statistical Computing, Vienna, Austria).

## 3. Results

Mothers who gave birth to live singletons (N = 7863), but whose children did not complete follow-up growth measurements (N = 3368) were slightly younger, were more likely to have lower educations and were less likely to have smoked during pregnancy. Response rates from individuals with Moroccan, Turkish and Surinamese-Indian backgrounds were comparable to Western ethnic groups, whereas response rates from Surinamese-African, Antillean and Ghanaian ethnic groups were lower.

The distributions of the various characteristics and child outcomes across the quartiles of the neighborhood SES are presented in Table 1. Neighborhoods with higher SES had lower perceived physical disorder and increased perceived safety. In such neighborhoods, maternal and pre-pregnancy BMIs were lower, fewer mothers had low levels of education, fewer mothers were from non-Western ethnicities, fewer mothers breastfed their infants for less than 3 months, and fewer mothers started solid foods before 4 months. Additionally, in neighborhoods with higher SES, child BMI and %BF were lower. The factors of interest were also analyzed according to the other three neighborhood factors (data not shown). The distribution of most covariates according to these neighborhood characteristics were as expected with the exception of satisfaction with green spaces. In neighborhoods with higher satisfaction with green spaces, maternal and pre-pregnancy BMIs were higher, more mothers were from non-Western ethnicities, and children had higher BMIs. 

Significant inverse relationships for both neighborhood SES and perceived safety were found for all outcomes in model 1 (Table 2), however for BMI and WHtR, these relationships were eliminated with the addition of maternal education and ethnicity (model 2). The associations with %BF were strongly attenuated but remained significant in model 2, but with the addition of the individual child history covariates (model 3) significance was lost. Significant positive relationships between satisfaction with green spaces and both %BF and WHtR were observed in model 1. As above, the relationship with WHtR was eliminated in model 2, while the relationship with %BF was eliminated in model 3. Finally, significant positive relationships were observed between physical disorder and both BMI and %BF in model 1, but were eliminated with the addition of maternal education and ethnicity (model 2).

ICCs were first calculated for the intercepts-only models and only a small percentage of the variation in outcomes was between postal code areas (1.4% of the variation in BMI, 3.0% of the variation in %BF and 1.9% of the variation in WHtR). In the models investigating neighborhood SES or perceived safety, the ICCs were attenuated somewhat for BMI and strongly for %BF when the neighborhood characteristic was included (model 1), but no additional changes were observed as familial and individual covariates were added. In the models investigating satisfaction with green spaces or physical disorder, inclusion of these neighborhood characteristics only slightly attenuated the ICC for BMI or %BF, whereas the addition of ethnicity and maternal education strongly attenuated the ICC, with no further changes in models 3 and 4. Thus variation in BMI and %BF between postal code areas was mostly explained by neighborhood SES, perceived safety, maternal education and ethnicity. The ICCs for WHtR did not change with the inclusion of any neighborhood, familial or individual covariates.

**Table 1:** Demographics and child outcomes per quartile of neighborhood socioeconomic status score.

Covariates of Interest	Number of Participants with Data	Quartile of Neighborhood Status Score			
	N	Low	Mid-Low	Mid-High	High
***Neighborhood characteristics***					
Mean status score (SD)	3469	−2.6 (0.8)	−1.3 (0.3)	−0.07 (0.5)	1.1 (0.3)
Mean perceived safety (SD)	3469	3.6 (0.1)	3.6 (0.2)	3.9 (0.2)	4.0 (0.09)
Mean satisfaction with green spaces (SD)	3469	3.5 (0.1)	3.6 (0.2)	3.5 (0.2)	3.5 (0.2)
Mean perceived physical disorder (SD)	3469	4.1 (0.7)	4.2 (0.8)	3.8 (0.8)	2.9 (0.5)
***Family characteristics***					
Maternal BMI (SD) (kg/m^2^)	2368	25.0 (4.9)	24.3 (4.4)	23.2 (3.8)	23.0 (3.6)
Overweight mother (BMI ≥ 25 kg/m^2^) (%)	2368	40.8	35.1	22.1	20.9
Paternal BMI (SD) (kg/m^2^)	2237	25.8 (4.2)	25.3 (3.3)	24.8 (2.9)	24.8 (2.8)
Overweight father (BMI ≥ 25 kg/m^2^) (%)	2237	53.1	48.4	42.1	43.1
Maternal Education	3451				
High-level (%)		29.3	41.6	59.1	70.5
Mid-level (%)		33.1	29.2	26.8	23.3
Low-level (%)		37.6	29.1	14.2	6.2
Non-Western ethnicity (%)	3469	60.4	40.2	18.2	13.5
***Individual history characteristics***					
Child age (SD) (years)	3469	5.8 (0.49)	5.7 (0.48)	5.6 (0.43)	5.6 (0.45)
Gender (% girls)	3469	49.0	50.4	45.4	54.9
Pre-pregnancy BMI (SD) (kg/m^2^)	3469	24.1 (4.6)	23.5 (4.3)	22.7 (3.8)	22.5 (3.3)
Overweight mother prior to pregnancy (BMI ≥ 25 kg/m^2^) (%)	3469	33.2	27.1	19.8	16.5
Smoking during pregnancy (% yes)	3468	12.1	14.8	11.8	5.0
Duration of exclusive breastfeeding	3469				
≥ 6 months (%)		15.3	18.4	16.3	14.5
3–6 months (%)		21.5	22.4	29.6	31.2
<3 months (%)		63.2	59.1	54.1	54.3
Introduction of solid foods before 4 months of age (%)	3469	6.7	5.7	5.2	4.8
***Child Outcomes***					
BMI standard deviation score (SD)	3469	0.14 (1.1)	0.10 (1.0)	−0.04 (0.98)	−0.1 (0.93)
Percentage body fat (%) (SD)	2465	22.5 (7.2)	21.2 (6.9)	20.0 (6.1)	19.6 (6.0)
Waist-to-height ratio (x100) (SD)	2497	45.3 (3.4)	45.2 (3.1)	45.2 (2.9)	45.0 (2.7)

In general, child BMI was positively associated with pre-pregnancy BMI, maternal smoking during pregnancy, maternal and paternal BMI and ethnicity. %BF was positively associated with child age, female gender, maternal and paternal BMI, low maternal education and non-Western ethnicity. Finally, WHtR was positively associated with maternal smoking during pregnancy and maternal BMI, and negatively associated with introduction of solid foods before 4 months, child age, female gender and non-Western ethnicity. Results of all analyses were similar in the sensitivity analysis excluding the combined postal code areas, although the relationships were somewhat attenuated. The sensitivity analysis using status scores from 2010 did not show any substantive changes from the primary analysis.

## 4. Discussion

Inverse relationships for neighborhood SES and perceived safety were observed with child adiposity, but only the relationships with child %BF remained significant, albeit strongly attenuated, after correction for maternal education and ethnicity. Positive relationships for satisfaction with green spaces and physical disorder with child adiposity were also observed, but these associations were eliminated with the inclusion of familial and individual child history covariates. Most of the unadjusted relationships that were observed appear to be a product of familial and individual characteristics that were unevenly distributed across neighborhoods, particularly uneven distributions of non-Western ethnic groups and mothers with low levels of education. Therefore, in Amsterdam, it appears that there is no direct influence of neighborhood characteristics on child adiposity in young children.

**Table 2:** Mixed model analyses for relationships between each neighborhood characteristic and child BMI, percent body fat and waist-to-height ratio.

Child Adiposity Outcome	Change in Outcome Variable per Unit Increase in Neighborhood Characteristic Score			
	Model 1 ^a^ (95%CI)	Model 2 ^b^ (95%CI)	Model 3 ^c^ (95%CI)	Model 4 ^d^ (95%CI)
**BMI ^§^**				
ICC * = 0.014				
Neighborhood SES	**−0.06 (−0.1, −0.03)**	−0.01 (−0.04, 0.02)	0.0005 (−0.03, 0.03)	0.008 (−0.03, 0.04)
ICC	0.0096	0.0092	0.0084	0.0093
Perceived neighborhood safety	**−0.5 (−0.7, −0.3)**	−0.1 (−0.3, 0.06)	−0.05 (−0.2, 0.1)	0.06 (−0.2, 0.3)
ICC	0.0073	0.0089	0.0083	0.0091
Satisfaction with green spaces	0.2 (−0.05, 0.5)	0.1 (−0.08, 0.3)	0.1 (−0.1, 0.3)	−0.002 (−0.3, 0.3)
ICC	0.013	0.087	0.080	0.0091
Physical disorder	**0.06 (0.003, 0.1)**	0.01 (−0.04, 0.06)	0.008 (−0.04, 0.05)	0.03 (−0.03, 0.08)
ICC	0.013	0.0097	0.0087	0.0096
**Percent body fat (%)**				
ICC * = 0.030				
Neighborhood SES	**−0.6 (−0.8, −0.4)**	**−0.2 (−0.4, −0.05)**	−0.2 (−0.4, 0.001)	−0.1 (−0.4, 0.06)
ICC	0.0065	0.0030	0.0034	0.0033
Perceived neighborhood safety	**−4.5 (−5.9, −3.2)**	**−1.4 (−2.7, −0.06)**	−0.9 (−2.2, 0.4)	−0.6 (−2.1, 0.9)
ICC	0.0034	0.0039	0.0044	0.0043
Satisfaction with green spaces	**2.2 (0.4, 4.2)**	**1.4 (0.04, 2.8)**	1.2 (−0.2, 2.6)	0.6 (−1.0, 2.2)
ICC	0.019	0.0040	0.040	0.0036
Physical disorder	**0.5 (0.04, 0.9)**	0.09 (−0.2, 0.4)	0.1 (−0.2, 0.4)	0.1 (−0.2, 0.5)
ICC	0.019	0.0056	0.0051	0.0048
**Waist-to-height ratio ^†^**				
ICC *^,^^‡^ = 0.019				
Neighborhood SES	**−0.1(−0.2, −0.04)**	−0.02 (−0.1, 0.09)	0.01 (−0.09, 0.1)	0.03 (−0.09, 0.1)
ICC **^‡^**	0.018	0.020	0.021	0.022
Perceived neighborhood safety ICC **^‡^**	**−1.0 (−1.7, −0.3)**	0.03 (−0.7, 0.8)	0.2 (−0.5, 1.0)	0.4 (−0.5, 1.2)
	0.019	0.020	0.021	0.021
Satisfaction with green spaces	**0.9 (0.1, 1.8)**	0.7 (−0.1, 1.4)	0.6 (−0.2, 1.3)	0.4 (−0.4, 1.3)
ICC **^‡^**	0.017	0.018	0.019	0.020
Physical disorder	0.2 (−0.03, 0.3)	0.04 (−0.1, 0.2)	0.04 (−0.1, 0.2)	0.07 (−0.1, 0.3)
ICC **^‡^**	0.020	0.021	0.022	0.022

**95%CI = 95% confidence interval. ^a^** Adjusted for gender and age in models with percent body fat and waist-to-height ratio as outcomes; **^b^** Additionally adjusted for maternal education and ethnicity; **^c^** Additionally adjusted for pre-pregnancy BMI, smoking during pregnancy, duration of exclusive breastfeeding and age at solid food introduction; **^d^** Additionally adjusted for maternal BMI and paternal BMI; **^§^** BMI converted to a standard deviation score, based on child age and gender using reference curves derived from ABCD study data; * ICC presented for complete cases (no missing data) to allow comparison between models. ICCs from total dataset are 0.022 for BMI, 0.051 for percent body fat and 0.013 for waist-to-height ratio; **^†^** Waist-to-height ratio multiplied by 100 for readability; **^‡^** ICC tested in transformed model.

The relationships between child adiposity outcomes and the individual history and family characteristics can be found in Table 3. 

**Table 3:** Mixed model analyses on relationships between individual and familial characteristics, and child BMI, percent body fat and waist-to-height ratio.

BMI ^§^	Change in Outcome Variable per Individual History or Family Characteristic
***Individual history characteristics***	
Pre-pregnancy BMI (kg/m^2^)	**0.03 (0.01, 0.04)**
Smoking during pregnancy	**0.3 (0.1, 0.4)**
Exclusive breastfeeding	
≥6 months	reference
3–6 months	0.02 (−0.1, 0.1)
<3 months	0.03 (−0.08, 0.1)
Age at introduction of solid foods	
≥4 months	reference
<4 months	−0.1 (−0.3, 0.05)
***Family characteristics***	
Maternal BMI (kg/m^2^)	**0.03 (0.01, 0.05)**
Paternal BMI (kg/m^2^)	**0.04 (0.02, 0.05)**
Maternal education	
High-level	reference
Mid-level	−0.07 (−0.2, 0.05)
Low-level	0.1 (−0.03, 0.3)
Non-Western ethnicity	**0.2 (0.04, 0.3)**
**Percent Body Fat**	
***Individual history characteristics***	
Pre-pregnancy BMI (kg/m^2^)	0.05 (−0.06, 0.2)
Smoking during pregnancy	0.6 (−0.3, 1.5)
Exclusive breastfeeding	
≥6 months	reference
3–6 months	−0.02 (−0.8, 0.7)
<3 months	0.4 (−0.3, 1.1)
Age at introduction of solid foods	
≥4 months	reference
<4 months	−0.7 (−1.9, 0.5)
Age	**2.5 (1.9, 3.1)**
Gender	
girl	**2.6 (2.1, 3.1)**
***Family Characteristics***	
Maternal BMI (kg/m^2^)	**0.2 (0.1, 0.3)**
Paternal BMI (kg/m^2^)	**0.1 (0.05, 0.2)**
Maternal education	
High-level	reference
Mid-level	−0.1 (−0.8, 0.5)
Low-level	**1.0 (0.05, 1.9)**
Non-Western ethnicity	**2.4 (1.6, 3.2)**
**Waist-to-height ratio ^†^**	
***Individual history characteristics***	
Pre-pregnancy BMI (kg/m^2^)	0.01 (−0.04, 0.06)
Smoking during pregnancy	**0.7 (0.2, 1.1)**
Exclusive breastfeeding	
≥6 months	reference
3–6 months	−0.1 (−0.5, 0.3)
<3 months	−0.06 (−0.4, 0.3)
Age at introduction of solid foods	
≥4 months	reference
<4 months	**−0.8 (−1.3, −0.2)**
Age	**−1.1 (−1.4, −0.8)**
Gender	
girl	**−0.4 (−0.6, −0.1)**
***Family characteristics***	
Maternal BMI (kg/m^2^)	**0.1 (0.06, 0.2)**
Paternal BMI (kg/m^2^)	0.03 (−0.01, 0.08)
Maternal education	
High-level	reference
Mid-level	−0.1 (−0.5, 0.2)
Low-level	0.3 (−0.2, 0.8)
Non-Western ethnicity	**0.5 (0.2, 0.9)**

**^§^** BMI converted to a standard deviation score, based on child age and gender using reference curves derived from ABCD study data; **^†^** Waist-to-height ratio multiplied by 100 for readability.

It is of interest that the relationships with child %BF remained significant after adjustment for maternal education and ethnicity. This suggests that neighborhood SES and perceived safety may have small influences on child %BF. The elimination of the relationship with the inclusion of individual history covariates and parental BMIs may imply that these covariates function as mediators. If the child was born in the same neighborhood within which he or she is currently living, and if the neighborhood influenced parental behaviors or contributed to parental overweight, these covariates could lie along the causal pathway to the child’s currently elevated %BF. However, these covariates could just as easily function as confounders particularly if the child was not born in the same neighborhood within which he or she is currently living. Thus, one cannot conclude with certainty that neighborhood SES and perceived safety indirectly influence %BF via individual history factors and parental overweight. The relationships observed after adjustment for maternal education and ethnicity may simply represent uneven distributions of the remaining individual and parental covariates. 

Uneven distributions of individual history and familial covariates likely explain the positive relationship between satisfaction with green spaces and %BF which unexpectedly remained significant after adjustment for maternal education and ethnicity. It is unlikely that neighborhoods with high satisfaction with green spaces increase %BF in children. In Amsterdam, the highest quality green spaces are found in neighborhoods where more non-Western ethnic groups and individuals of lower SES reside, and there is a higher prevalence of overweight in both adults and children in these neighborhoods. In this study, mothers in these neighborhoods had both higher pre-pregnancy and current BMIs which likely contributed to the higher %BF in the children.

### 4.1. Strengths and Limitations

We were limited in the current study by not having information available on how long families had been living within a given neighborhood. If a family moved to a neighborhood only recently, it is less likely that the neighborhood factors could contribute to child adiposity. Additionally, without this data, it is difficult to elucidate whether the individual history and familial characteristics included in the analyses functioned as mediators or confounders. 

Selection bias may also be present in this study because of lower participation rates in the ABCD cohort from non-Western ethnic groups and lower income individuals [36,49]. Within the current study sample, fewer women with low levels of education and from non-Western ethnicities, specifically African backgrounds, participated in the follow-up measurements on their 5–6 year old child. The underrepresentation of individuals with African backgrounds reduces the generalizability of the results to these ethnic groups. In previous analyses of the ABCD cohort, despite the selective non-response, selection bias in pregnancy outcomes was minimal [49]. It is unknown to what extent selection bias affects the longer term outcomes investigated in this study, but it would likely reduce the observed effect sizes due to the greater participation by lower risk individuals. 

In the WoON survey, the response rate was 63% [50]. Lower response rates were observed in non-Western immigrant groups (47%) and among households with lower incomes (52%) [50]. Selective response may have affected the measurement of neighborhood characteristics in our study. However, it seems unlikely that this would have seriously biased the assessment of the differences between neighborhoods. 

The timing of the anthropometric measurements may also be problematic. Most children experience an adiposity rebound at about 6 years of age where the BMI drops to a minimum and then increases again. Therefore when measured in this study, some children may be in a stage of declining BMI and others may be experiencing an increasing BMI. Therefore, we utilized BMI standard deviation scores to account for age at measurement to address the stage of adiposity rebound. Our results may still be valid beyond 6 years of age because higher BMI values earlier in life, including during the adiposity rebound, are predictors for overweight later in childhood [5,51]. 

Finally, there were limitations to the BIA measurements. Although we utilized a corrected and validated equation for BIA measurement in 4–7 year old children, there is still the potential for ethnic differences which are not addressed by this equation. The ideal method of measuring child %BF would be dual-energy x-ray absorptiometry; however, such measurements were not possible in this large scale cohort. 

The current study benefited from the use of separate data for neighborhood characteristics rather than the opinions of the parents in the ABCD study. This allows for an understanding of how the general community, rather than individuals, sees the neighborhood and to determine if a community level intervention would be appropriate. This study also benefited from having data available on %BF and WHtR, thus allowing analysis of important adiposity outcomes other than BMI. 

### 4.2. Previous Research

Only a few studies [16,17,18,19,22,25,26] have specifically focused on preschool children and the results of the current study are in line with most of this previous research. In two American studies, no relationships were observed between neighborhood safety and overweight in 3–5 year old children [17,18]. An Australian study [19] observed no relationships between parental perceptions of neighborhood safety and overweight in 5–6 year old children, but a positive association was observed for parental concerns over traffic safety in 10–12 year old children. Another Australian study [16] found that neighborhood SES was not significantly related to BMI in 4–5 year old children, whereas the relationship was significant in older children. Finally, an American study [26] observed an inverse relationship between objective traffic safety measures and body fat in 2–5 year old children, but no association was observed with BMI. Of note, the models in this study did not include individual child history and parental BMI covariates, thus it is unknown whether the observed relationship with fat mass would have remained significant after adjustment for these variables. Based on the previous research and the findings of the current study, it is possible that preschool children who are exposed to neighborhoods with low SES and low perceived safety have increased fat mass, but that differences in BMI only become apparent at an older age. This emphasizes the importance of investigating %BF as an outcome in future studies on this age group.

The results of the current study were inconsistent with previous North American research on the relationships between land-use or quality of green spaces with child adiposity [22,25,26]. These previous studies observed relationships for better walkability and less urbanicity with reduced adiposity in young children, whereas no relationship was observed in the current study. These discrepancies may be in part due to the objective measurement of green spaces in the previous research *versus* the subjective measurement in the current study. Additionally, in North America most of the best green spaces are in the suburban areas where higher SES individuals reside. In contrast, as previously discussed, the greenest neighborhoods in Amsterdam are mostly populated by lower SES individuals and non-Western ethnicities and, as such, children in these neighborhoods are at risk for increased adiposity Therefore, it would appear that in the Amsterdam setting, the quality of green spaces is not an important factor in reducing adiposity in 5–6 year old children.

### 4.3. Implications

This study contributes to the current literature by investigating adiposity outcomes other than BMI that are important predictors of future health. Additionally, this study increases knowledge of how neighborhood factors influence child adiposity in a European setting, for which there are far fewer studies than in North America. Although the results suggest that there is no direct influence of neighborhood characteristics on adiposity in young children, improving neighborhood SES and safety may improve the lifestyle behaviors of parents, which could subsequently improve the health of their children. Community based intervention studies could help to clarify this hypothesis. 

## 5. Conclusions

In general, child adiposity at age 5–6 years was not independently associated with neighborhood characteristics, although a small relationship may exist between child %BF and both neighborhood SES and perceived safety. Ultimately at this young age, familial and individual factors probably play a more important role in influencing child adiposity than neighborhood characteristics.
